# Experimental and analytical geodetic accuracy assessment of terrestrial laser scanning for deformation monitoring under static and dynamic conditions

**DOI:** 10.1038/s41598-026-53004-6

**Published:** 2026-06-13

**Authors:** Ashraf A. A. Beshr, Talal Obaid Alshammari, Ismail Zaher, Ashraf G. Shehata

**Affiliations:** 1 Public Works Department, Faculty of Engineering, Mansoura University, Mansoura, 35516 Egypt; 2 Department of Civil Engineering, College of Engineering, Jouf University, 72388 Sakaka, Saudi Arabia; 3 Civil Engineering Department, Faculty of Engineering, Delta University, Mansoura, Egypt

**Keywords:** Terrestrial laser scanning (TLS), Deformation monitoring, Vibration effects, Structural health monitoring, Oil storage tanks, Engineering, Optics and photonics

## Abstract

Recent advances in three-dimensional (3D) measurement technologies have significantly improved the capability of monitoring the structural deformation. Among these technologies, terrestrial laser scanning (TLS) has emerged as a powerful geodetic tool capable of rapidly acquiring dense 3D point clouds with millimeter-level accuracy. However, the reliability of TLS for high-precision deformation monitoring requires comprehensive experimental and analytical validation and assessment under both static and dynamic measurement conditions. This study presents a detailed experimental and analytical evaluation of the geometric accuracy of TLS observations for structural deformation monitoring. A series of controlled experiments were conducted to assess the accuracy of coordinates, distances and angular measurements derived from TLS by comparing them with reference observations obtained using a high-precision total station. The capability of TLS to detect small structural displacements was also investigated through controlled horizontal, vertical and inclined movements measured using a digital accurate vernier device. Furthermore, the influence of tripod-induced vibrations on TLS measurement accuracy was examined under different vibration frequencies and amplitudes in order to simulate dynamic field conditions. The practical applicability of TLS in structural health monitoring was also demonstrated through a real industrial case study involving the geometric assessment and inclination analysis of a vertical cylindrical oil storage tank located in Ras – Gharib city, Egypt. The experimental results indicate that TLS can detect structural displacements with discrepancies ranging between 0.78 and 2.16 mm compared with reference measurements, while vibration effects can introduce distance variations of up to 4.1 mm and horizontal angular deviations reaching approximately 100 arc-seconds under several vibration conditions. These results provide practical insights into the capabilities and operational limitations of TLS technology and support its effective application in structural deformation monitoring and precision engineering surveying.

## Introduction

Monitoring the structural deformation is an essential task in engineering surveying and structural health monitoring, particularly for critical infrastructure such as bridges, high-rise buildings, industrial plants, and oil storage tanks^1^. Detecting small geometric changes in such structures requires highly accurate geodetic measurement techniques which are capable of providing reliable spatial accurate information. Recent advances in three-dimensional sensing technologies have significantly improved the ability to monitor structural geometry and deformation processes^2,3^. Traditionally, deformation monitoring has been performed using conventional surveying instruments such as total stations, precise leveling, close range photogrammetry and Global Navigation Satellite Systems (GNSS).

In recent years, terrestrial laser scanning (TLS) has emerged as a powerful three-dimensional measurement technology capable of rapidly acquiring dense point clouds that represent the geometry of objects with high spatial resolution and accuracy. TLS instruments use laser ranging techniques to determine the three-dimensional coordinates of millions of points on the surface of an object within a short acquisition time^4^. This capability allows engineers and surveyors to capture complete geometric information about structures, enabling detailed deformation analysis and structural assessment. The advantages of TLS include rapid 3D data acquisition, high point density though points clouds, non-contact geodetic measurements, and the ability to capture complex structural geometries and details that are difficult to observe using traditional surveying techniques^5,6^.

Due to these advantages, TLS has been increasingly applied in various engineering projects and geodetic fields including infrastructure inspection, deformation monitoring, industrial plant modeling and structural health monitoring. Recent studies and researchers have demonstrated the potential of TLS for monitoring the structural deformation of different engineering structures and analyzing geometric changes in its elements. For example, Khoshelham and Zhang^3^ highlighted the growing role of TLS in geospatial applications and discussed recent technological developments that improved its measurement capabilities. Similarly^7^, and Piekarczuk^8^, reviewed recent advances in three-dimensional laser scanning technologies and their applications in engineering surveying and geomatic applications. In addition, Papadopoulou and Kavadias^9^ reported that LiDAR-based monitoring systems provide valuable tools for structural health monitoring due to their ability to capture detailed spatial data and information of large structures condition assessment. Other studies have also demonstrated the effectiveness of using TLS in structural damage detection and deformation analysis^10–14^. To distinguish between total station observations and TLS-derived measurements in structural monitoring, Table 1 provides a comparative summary that highlights differences in measurement principles, achievable accuracies, data acquisition procedures, and practical advantages and limitations of each technique^15^ and^1^.

**Table 1:** Comparison between total station and TLS measurements in structural monitoring.

Comparison criteria	Total station	Terrestrial laser scanner (TLS)
Measurement principle	Point-based measurements using angles and distances	Laser-based acquisition of dense 3D point clouds
Data collected and sampling rate	Point by point coordinates	Cloud of thousands of points coordinates
Creation of 3D defect model	Cannot create	Available
Accuracy	Very high accuracy at selected points	High accuracy over full surfaces
Spatial coverage	Limited to selected monitoring points	Full-field surface coverage
Devices availability	Available	Available
Limitations	May miss localized deformation between points	Sensitive to noise, vibration, and requires more processing
Time required	A lot of time to finish detecting all details	Takes a little time to complete all details with high accuracy

Despite these developments, the application of TLS in high-precision deformation monitoring still faces several challenges. The accuracy of TLS observations may be influenced by various factors such as scanning distance, angular resolution, environmental conditions and instrument stability during measurements. In particular, vibration effects caused by tripod instability, nearby machinery, or environmental disturbances may significantly influence the accuracy of measured coordinates, distances and angles. Although several studies have investigated TLS accuracy and calibration methods^16,17^, comprehensive experimental investigations that simultaneously evaluate the coordinate accuracy, deformation detection capability, and vibration influence under controlled conditions remain limited. Therefore more studies are required to actually investigate the accuracy assessment of TLS under static and dynamic conditions.

TLS has also shown also tremendous promise in post-earthquake applications, particularly for quick damage assessment, structural deformation monitoring, and detecting ground movements such as settlements and landslides. Its ability to generate dense and accurate 3D data makes it an invaluable tool for post-disaster surveying and structural state assessment. Recent seismic studies highlight the significance of improved monitoring approaches for damage assessment and decision-making in civil and earthquake engineering^18–21^. These applications emphasise the necessity to evaluate TLS performance and stability in dynamic situations.

Furthermore, many previous studies have focused mainly on theoretical analysis or laboratory validation without providing practical demonstrations in real industrial environments. For critical engineering structures such as cylindrical oil storage tanks, accurate monitoring of geometric parameters and structural inclination is essential to ensure operational safety and structural stability^7,22,23^. However, the practical performance of TLS in detecting such geometric changes under realistic field conditions (dynamic conditions) still requires further studies and investigation^24,25^.

Therefore, the primary objective of this paper is to provide a comprehensive experimental and analytical evaluation of terrestrial laser scanning accuracy for structural deformation monitoring applications in static and dynamic conditions. The study includes controlled experimental tests to assess coordinate accuracy, distance and angular measurement precision, and the capability of TLS to detect small structural displacements. Total station and digital vernier observations were used as reference measurements for the comparative accuracy evaluation because their measurement uncertainties are much lower than those of the terrestrial laser scanner; additionally, short observation distances were used to reduce reference measurement errors, and the 0.02 mm digital vernier resolution offered dependable displacement control. In addition, the influence of tripod-induced vibration on TLS observations is investigated under different vibration levels to simulate dynamic field conditions. Finally, a real industrial case study is presented to evaluate the applicability of TLS in determining the geometric parameters and inclination of a vertical cylindrical oil storage tank located in Ras Gharib city, Egypt. Therefore, the main contributions (novelty) of this research can be summarized as follows:

1. A comprehensive experimental framework is developed to evaluate the geometric accuracy of TLS observations under different scanning distances and angular resolutions and compare with accurate Total Station observations.
2. The ability of TLS to detect millimeter-level structural displacements is experimentally investigated using controlled deformation tests and comparing with digital vernier.
3. The influence of tripod vibration on TLS measurement accuracy is quantitatively analyzed under different vibration frequencies and amplitudes.
4. The study demonstrates the practical applicability of TLS in structural health monitoring through a real industrial case study involving the geometric analysis and inclination assessment of a large oil storage tank.

## Determination of point coordinates and its accuracy from TLS measurements

Terrestrial laser scanning (TLS) determines the three-dimensional coordinates of object points by measuring the slope distance and the corresponding horizontal and vertical angles between the scanner and the target point. Modern TLS instruments operate using laser ranging techniques combined with precise angular measurements to compute the spatial coordinates of millions of points on object surfaces (points clouds) within a short acquisition time (scanning time). These measurements form dense point clouds that accurately represent the geometry of scanned structures^4,26^.

In principle, the determination of point coordinates from TLS observations follows the same geometric concept used in reflectorless total station measurements, where the coordinates of an observed point are derived from the measured slope distance and angular directions relative to the instrument position. Assuming that the scanner coordinate system is located at the origin of the reference coordinates frame, the spatial coordinates of any observed point (i) can be determined from the measured slope distance (S_i_), horizontal angle (α_i_), and vertical angle (γ_i_) as follows^7^:

1 $$\left. \begin{aligned} {\mathrm{X}}_{{\mathrm{i}}} & = {\mathrm{S}}_{{\mathrm{i}}} \;\cos \gamma _{{\mathrm{i}}} ,\;\sin \;\alpha _{i} ; \\ {\mathrm{Y}}_{{\mathrm{i}}} & = {\mathrm{S}}_{{\mathrm{i}}} \;\cos \gamma _{{\mathrm{i}}} ,\;\sin \;\alpha _{{\mathrm{i}}} ; \\ {\mathrm{Z}}_{{\mathrm{i}}} & = {\mathrm{S}}_{{\mathrm{i}}} \;\sin \;\gamma _{{\mathrm{i}}} \\ \end{aligned} \right\}$$

where: (S_i_) represents the measured slope distance between the scanner and the observed point, (α_i_) and (γ_i_) denote the horizontal and vertical angles, respectively.

The accuracy of the derived point coordinates depends on the precision of the measured distance and angular observations. In TLS measurements, the accuracy is influenced by several factors including instrument specifications, scanning distance, angular resolution and environmental conditions during the scanning process^7,27^. Therefore, the propagation of measurement errors from the basic observations to the computed coordinates must be considered when evaluating the reliability of TLS observations.

Using the law of error propagation, the standard deviations of the coordinates (X, Y, Z) can be expressed as functions of the standard deviations of the measured distance and angles. Let (*m*_*S*_), (*m*_*α*_), and (*m*_*γ*_) denote the standard deviations of the slope distance, horizontal angle and vertical angle measurements, respectively. By differentiating the coordinate equations (Eqs. (1)) with respect to the observed parameters, the accuracy of the point coordinates can be estimated through the multivariate error propagation model. This approach provides a theoretical estimation of coordinate accuracy based on the instrumental measurement precision and observation geometry as following:

2 $$\left. {\begin{array}{*{20}l} {m_{X}^{2} = \left( {\frac{X}{{\sqrt {X^{2} + Y^{2} + Z^{2} } }}} \right)^{2} m_{S}^{2} + (Y)^{2} m_{\alpha }^{2} + \left( {\frac{ZX}{{\sqrt {X^{2} + Y^{2} } }}} \right)^{2} m_{\gamma }^{2} ;} \hfill \\ {m_{Y}^{2} = \left( {\frac{Y}{{\sqrt {X^{2} + Y^{2} + Z^{2} } }}} \right)^{2} m_{S}^{2} + (X)^{2} m_{\alpha }^{2} + \left( {\frac{ZY}{{\sqrt {X^{2} + Y^{2} } }}} \right)^{2} m_{\gamma }^{2} ;} \hfill \\ {m_{Z}^{2} = \left( {\frac{Z}{{\sqrt {X^{2} + Y^{2} + Z^{2} } }}} \right)^{2} m_{S}^{2} + \left( {\sqrt {X^{2} + Y^{2} } } \right)^{2} m_{\gamma }^{2} .} \hfill \\ \end{array} } \right\}$$

The obtained accuracy estimates are particularly important in applications involving structural deformation monitoring, where the detection of small geometric changes requires reliable coordinate determination at the millimeter level. Consequently, both theoretical accuracy estimation and experimental validation are necessary to evaluate the performance of TLS systems in high-precision engineering surveys. Similar analytical approaches have been widely applied in TLS calibration and deformation monitoring studies to assess the reliability of derived spatial coordinates.

## Experimental assessment of TLS accuracy under different scanning distances and resolutions

**Fig. 1:**
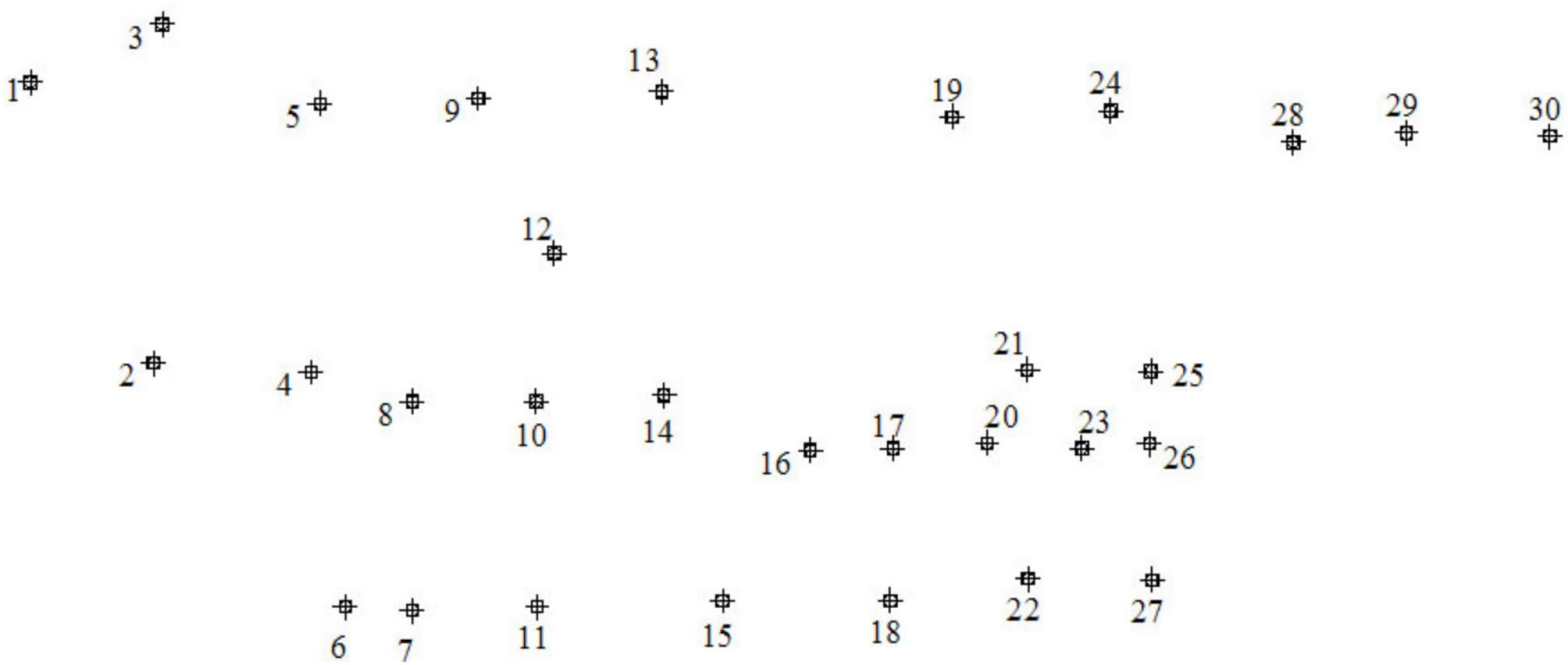
Spatial distribution of the 30 reflective targets on the vertical flat wall.

To evaluate the geometric accuracy of terrestrial laser scanning (TLS) observations, a controlled experimental study was conducted in which TLS measurements were compared with reference observations obtained from a high-precision total station. Such comparative experiments are commonly used in geodetic studies to assess the accuracy and reliability of laser scanning measurements. For this purpose, thirty reflective targets were fixed on a vertical flat wall to provide a stable and well-defined reference surface. Each target was carefully mounted and clearly identifiable in both the TLS point cloud and the total station observations. The targets were distributed across the wall to ensure an adequate geometric configuration for evaluating coordinate accuracy and derived geometric parameters. The spatial distribution of the observed targets is illustrated in Fig. 1.

The reference coordinates of all target points were determined using a Trimble Zeiss 3305 DR total station operating in reflectorless mode, which has a nominal distance measurement accuracy of approximately 5 mm. The total station was positioned at a relatively short distance from the wall in order to minimize potential systematic effects related to atmospheric refraction, measurement geometry and instrument setup errors. The obtained coordinates from total station observations were considered as reference values for the subsequent accuracy assessment.

TLS observations were carried out using a Riegl LMS-Z420i terrestrial laser scanner. According to the manufacturer specifications, the instrument provides a single distance measurement accuracy of approximately 5 mm and angular accuracies of about 6″ and 7″ for vertical and horizontal angle measurements, respectively. These characteristics make the instrument suitable for engineering surveying and structural monitoring applications.

To investigate the influence of scanning geometry on measurement accuracy, three different distances between the scanner and the observed wall were considered: 14.848 m, 20.714 m and 26.491 m. For each distance, scanning was performed using three different angular resolutions: 0.035°, 0.015°, and 0.007°. This experimental design resulted in a total of nine independent scanning datasets, enabling a comprehensive assessment of the combined effects of scanning distance and angular resolution on the accuracy of TLS measurements. The experiments were conducted under normal environmental conditions during daytime measurements to minimize potential effects caused by temperature fluctuations or atmospheric disturbances. This controlled experimental setup allowed a systematic evaluation of the influence of scanning distance and resolution on the accuracy of TLS observations in engineering surveying applications.

The acquired TLS point clouds were processed using RiSCAN PRO software. The processing workflow included automatic detection of reflective targets, extraction of their spatial coordinates within the scanner coordinate system, and transformation of the results to the reference coordinate frame defined by the total station observations. The exterior orientation parameters of each scan were also computed during the processing stage to ensure accurate alignment between TLS data and the reference measurements.

To quantify the accuracy of TLS observations, several statistical indicators were evaluated. First, the coordinate differences between TLS-derived points and total station reference coordinates were computed for the three coordinate components (X), (Y), and (Z). Second, statistical measures including the root mean square error (RMSE) and standard deviation were calculated to evaluate the positional accuracy of TLS observations under different scanning configurations. In addition, inter-point distances derived from TLS coordinates were compared with those obtained from total station measurements in order to assess the relative geometric accuracy of the scanning results.

Table 2 presents the minimum and maximum standard deviations of the point coordinates derived from total station observations and from the nine TLS scanning configurations. The results clearly indicate that the coordinate accuracy obtained from the total station is higher than that derived from TLS measurements, particularly in the horizontal components (X and Y). For the total station observations, the standard deviations of the coordinates range between 2.76 and 4.59 mm for the X component and between 1.96 and 4.28 mm for the Y component, while the vertical component Z shows smaller values ranging from 0.61 to 1.21 mm.

**Table 2:** Minimum and maximum standard deviations of point coordinates obtained from total station and TLS observations under different scanning distances and angular resolutions.

Observation Type	Angular resolution (°)	Standard deviations of points coordinates, mm							
		X		Y		Z		R	
		Min	Max	Min	Max	Min	Max	Min	Max
Total station	–	2.76	4.59	1.96	4.28	0.61	1.21	5.05	5.23
TLS (14.848 m)	0.035°	8.54	12.25	0.78	8.78	0.51	2.14	12.32	12.35
	0.015°	8.55	12.24	0.72	8.77	0.51	2.13	12.32	12.36
	0.007°	8.55	12.26	0.72	8.77	0.51	2.13	12.32	12.36
TLS ( 20.714 m)	0.035°	11.68	12.41	0.91	4.15	0.71	1.76	12.46	12.49
	0.015°	11.71	12.42	0.91	4.14	0.70	1.67	12.47	12.49
	0.007°	11.69	12.41	0.90	4.11	0.71	1.68	12.46	12.49
TLS ( 26.491 m)	0.035°	8.54	12.25	0.72	8.77	0.509	2.13	12.32	12.35
	0.015°	8.54	12.26	0.73	8.77	0.51	2.13	12.32	12.36
	0.007°	8.55	12.26	0.74	8.77	0.51	2.13	12.32	12.35

In contrast, the TLS observations exhibit larger standard deviations in the coordinate components due to the influence of scanning geometry, angular resolution, and instrumental characteristics. For example, at a scanning distance of 14.848 m, the standard deviation of the X component ranges between approximately 8.54 mm and 12.26 mm, while the Y component varies between about 0.72 mm and 8.78 mm depending on the selected scanning resolution. Similar trends are observed for the other scanning distances. The results also indicate that the accuracy of TLS-derived coordinates is influenced by both the scanning distance and the selected angular resolution. In general, higher angular resolutions tend to produce slightly more stable coordinate estimates, while increasing the distance between the scanner and the object leads to a gradual degradation in coordinate accuracy. This behavior is consistent with previously reported studies on TLS accuracy, which show that the geometric configuration of the scanning setup plays a significant role in the precision of derived spatial coordinates. Overall, the obtained results confirm that although TLS provides slightly lower coordinate accuracy compared with total station observations, it achieves millimeter-level precision that is sufficient for many engineering applications involving structural monitoring and geometric analysis.

## Accuracy assessment of TLS observations based on Inter-Point distances and angles

In addition to evaluating the accuracy of the individual point coordinates, the relative geometric accuracy of TLS observations can also be assessed by analyzing the distances between selected pairs of observed points. This approach is widely used in deformation monitoring studies because inter-point distances and angles are less sensitive to systematic coordinate shifts and provide a reliable indicator of the internal geometric consistency of the measured dataset^28–30^.

To further evaluate the geometric consistency of the obtained coordinates, the distances between selected pairs of observed points were computed using the derived coordinates from both total station and TLS observations. The distance between the two observed points (i) and (j) (reflective marks) can be determined from their spatial coordinates using the following equation:

3 $$D = \sqrt {\left( {X_{j} - X_{i} } \right)^{2} + \left( {Y_{j} - Y_{i} } \right)^{2} + \left( {Z_{j} - Z_{i} } \right)^{2} }$$

where Xi, Yi, Zi and Xj, Yj, Zj represent the coordinates of the first and second points defining the measured line.

The accuracy of the computed distance can be estimated using the law of error propagation by differentiating Eq. (3) with respect to the coordinate components. After simplification, the standard deviation of the distance can be expressed as follows:

4 $$\begin{aligned} m_{D}^{2} & = \left( {\frac{{X_{j} - X_{i} }}{D}} \right)^{2} m_{{X_{j} }}^{2} + \left( {\frac{{X_{i} - X_{j} }}{D}} \right)^{2} m_{{X_{i} }}^{2} + \left( {\frac{{Y_{j} - Y_{i} }}{D}} \right)^{2} m_{{Y_{j} }}^{2} + \left( {\frac{{Y_{i} - Y_{j} }}{D}} \right)^{2} m_{{Y_{i} }}^{2} \\ & \quad + \left( {\frac{{Z_{j} - Z_{i} }}{D}} \right)^{2} m_{{Z_{j} }}^{2} + \left( {\frac{{Z_{i} - Z_{j} }}{D}} \right)^{2} m_{{Z_{i} }}^{2} \\ \end{aligned}$$

where $m_{D}$ represents the standard deviation of the computed distance, while $m_{{x_{i} }} ,m_{{y_{i} }} ,m_{{z_{i} }} ,m_{{x_{j} }} ,m_{{y_{j} }} ,m_{{z_{j} }}$ are the standard deviations of the coordinate components of points $i$ and $j$, respectively.

For this purpose, several distances between the observed targets (reflective marks) were computed for both the total station reference coordinates and the coordinates derived from the TLS point clouds. The distances obtained from the total station observations were considered as reference values due to the high precision of the instrument. The corresponding distances calculated from TLS coordinates were then compared with the reference distances to evaluate the relative accuracy of the TLS measurements. The calculated distances and their corresponding standard deviations obtained from total station and TLS observations are summarized in Table 3.

**Table 3:** Minimum, maximum, and average inter-point distances and their standard deviations derived from total station and TLS observations at different scanning distances and resolutions.

Parameter	Total station	Distance 14.848 m			Distance 20.714 m			Distance 26.491 m		
		0.0350°	0.0150°	0.0070°	0.0350°	0.0150°	0.0070°	0.0350°	0.0150°	0.0070°
Distances between points (m)										
Min	1.100	1.118	1.107	1.101	1.123	1.101	1.105	1.118	1.107	1.101
Max	12.225	12.245	12.250	12.241	12.264	12.239	12.234	12.245	12.250	12.241
Average	6.381	6.393	6.394	6.390	6.410	6.389	6.391	6.393	6.394	6.390
S.D. for distances between points (mm)										
Min	2.934	4.256	4.299	4.232	3.695	3.657	3.625	4.256	4.299	4.232
Max	4.972	9.989	9.983	9.986	5.369	5.351	5.283	9.989	9.983	9.986
Average	4.480	7.572	7.582	7.573	4.399	4.387	4.357	7.572	7.582	7.573

Table 3 summarizes the minimum, maximum and average distances between selected pairs of observed points (reflective marks) together with their corresponding standard deviations derived from both total station and TLS observations under different scanning distances and angular resolutions. The results provide an additional assessment of the relative geometric accuracy of the measured point coordinates.

The distances computed from the total station observations range between 1.100 and 12.225 m, with an average distance of approximately 6.381 m. The corresponding standard deviations of the measured distances vary between 2.934 and 4.972 mm, with an average value of about 4.480 mm, reflecting the high precision typically achieved by total station measurements. In comparison, the distances derived from TLS observations exhibit slightly larger standard deviations due to the inherent characteristics of laser scanning measurements and the influence of scanning geometry. For example, at a scanning distance of 14.848 m, the average standard deviation of the computed distances is approximately 7.57 mm, while the maximum standard deviation reaches about 9.99 mm. Similar accuracy levels are observed at the longer scanning distance of 26.49 m, where the average standard deviation remains close to 7.57 mm.

Interestingly, the TLS observations obtained at a scanning distance of approximately 20.714 m show improved relative distance accuracy, with an average standard deviation of about 4.38 mm, which is comparable to the accuracy obtained from total station observations. This behavior may be attributed to a more favorable scanning geometry and incidence angle between the laser beam and the observed surface, which can influence the precision of laser ranging measurements.

Overall, the results indicate that the relative distance accuracy obtained from TLS observations generally ranges between approximately 4 mm and 8 mm depending on the scanning configuration. These values are consistent with the expected performance of terrestrial laser scanning systems reported in previous studies^3,6,31^. The obtained results confirm that TLS observations are capable of preserving the relative geometric relationships between measured points with millimeter-level precision, which is sufficient for many engineering surveying and structural monitoring applications.

**Fig. 2:**
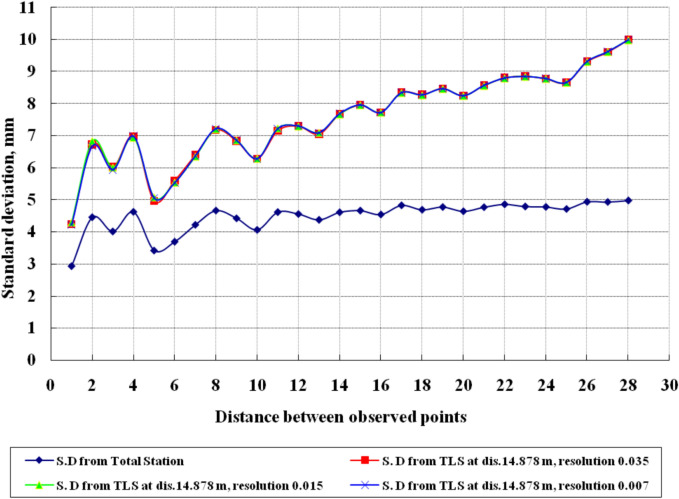
Comparison of the distance accuracy resulted from the observed points coordinates from total station and TLS observations at different resolution at distance 14.878 m.

**Fig. 3:**
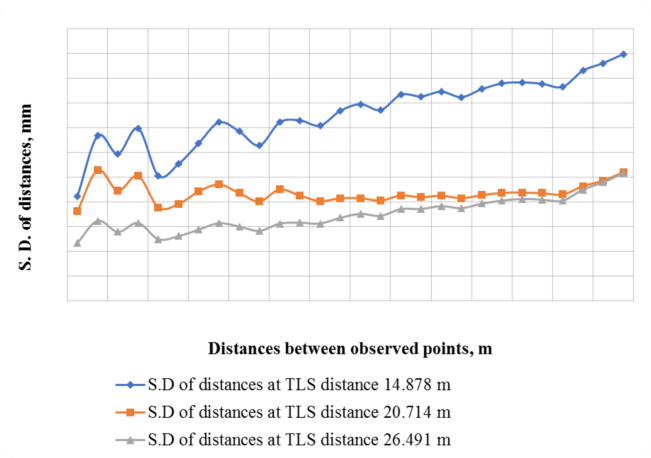
Comparison of the standard deviations of the calculated distances between points resulted from TLS observations at resolution 0.007°.

**Fig. 4:**
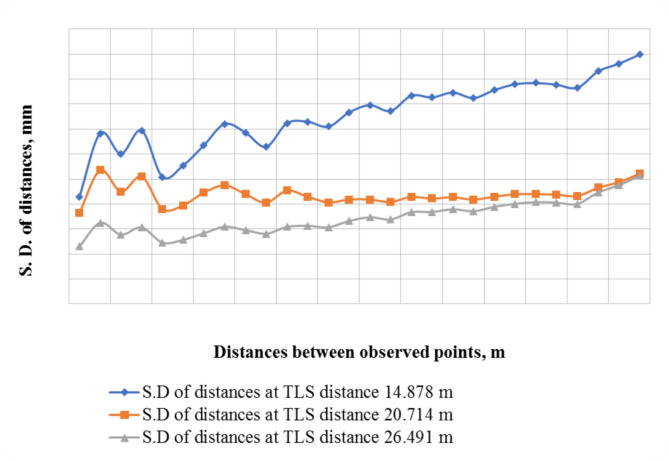
Comparison the standard deviations of the calculated distances between points resulted from TLS observations at resolution 0.015°.

**Fig. 5:**
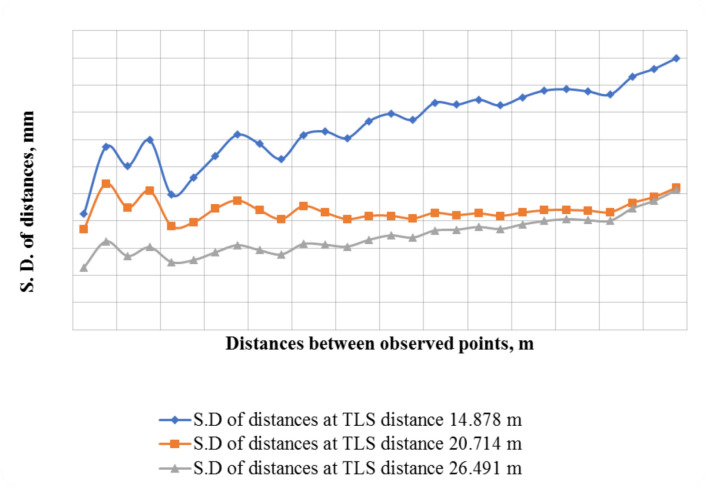
Comparison the standard deviations of the calculated distances between points resulted from TLS observations at resolution 0.035°.

To further illustrate the variation of inter-point distance accuracy under different scanning configurations, the results presented in Table 2 are graphically represented in Figs. 2, 3, 4 and 5.

As illustrated in Figs. 2, 3, 4 and 5, the graphical representation of the results confirms the trends observed in Table 2. The TLS-derived distances show slightly larger variability compared with the total station measurements, while still maintaining millimeter-level accuracy. The figures also highlight the influence of scanning distance and angular resolution on the precision of the derived distances.

This section investigates the accuracy of horizontal and vertical angle measurements derived from TLS observations under different scanning distances (14.878 m, 20.714 m, and 26.491 m) and angular resolutions (0.007°, 0.015°, and 0.035°), using total station observations as reference measurements. The objective is to analyze how scanning distance and angular resolution influence the precision of angular measurements between observed points in TLS data.

**Fig. 6:**
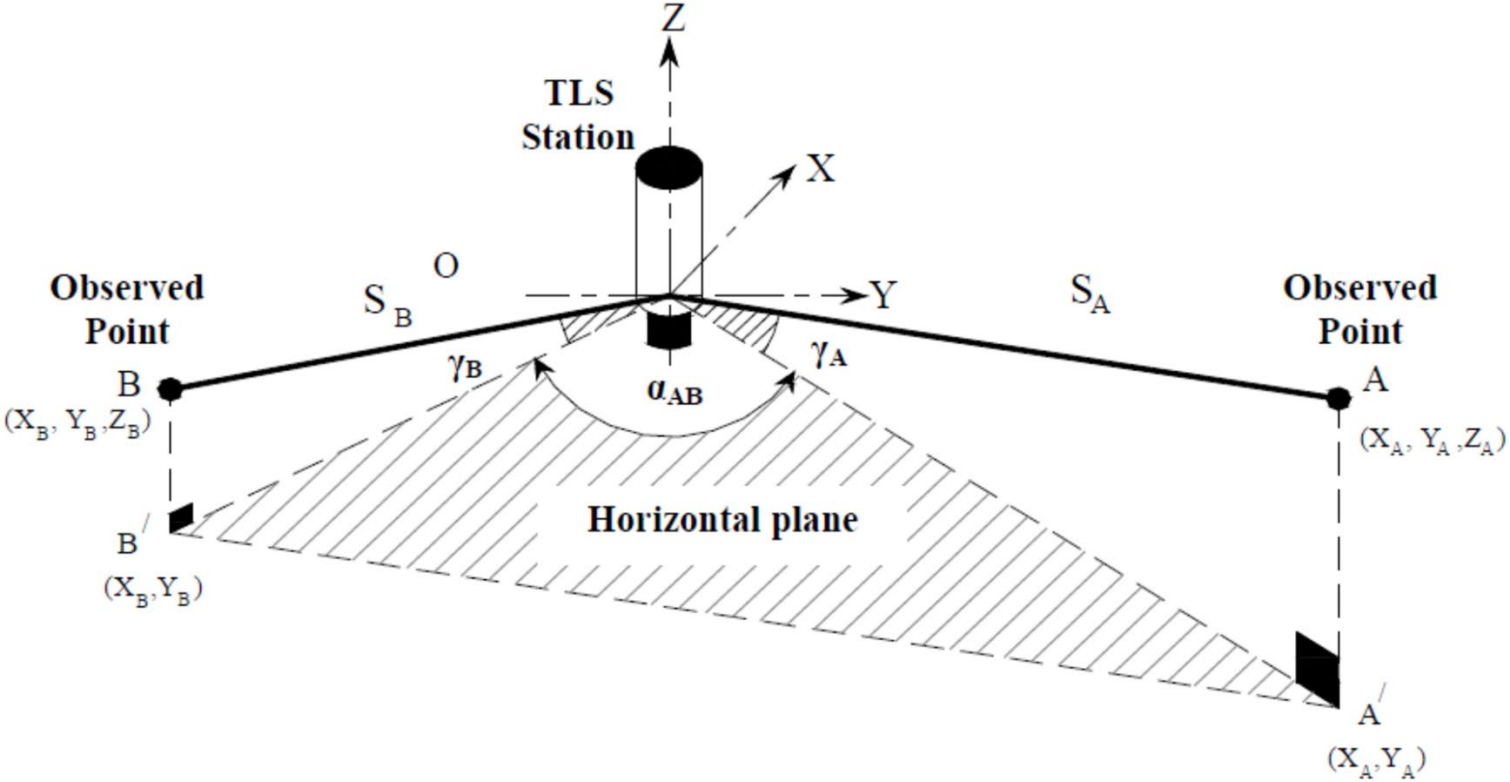
Geometry used for calculating the horizontal and vertical angles between two scanned points in TLS observations.

As shown in Fig. 6, the horizontal angle α_AB_ can be calculated from the geometry of the horizontal triangle OA^/^B^/^ as follows:

5 $$\cos \alpha_{AB} = \frac{{OA^{/ 2} + OB^{/ 2} - A^{/} B^{/ 2} }}{2OA\;OB}.$$

If the coordinates of laser scanner position equal (0, 0, and 0) at any scan, the Eq. (5) can be reconstructed by using the point’s coordinates as following:

6 $$\cos \alpha_{AB} = \frac{{X_{A} X_{B} + Y_{A} Y_{B} }}{{\sqrt {X_{A}^{2} + Y_{A}^{2} } \sqrt {X_{B}^{2} + Y_{B}^{2} } }} ,$$

The accuracy of horizontal angle has the form:

7 $$m_{{\alpha_{AB} }}^{2} = \left( {\frac{{\partial \alpha_{AB} }}{{\partial X_{A} }}} \right)^{2} m_{{X_{A} }}^{2} + \left( {\frac{{\partial \alpha_{AB} }}{{\partial Y_{A} }}} \right)^{2} m_{{Y_{A} }}^{2} + \left( {\frac{{\partial \alpha_{AB} }}{{\partial X_{B} }}} \right)^{2} m_{{X_{B} }}^{2} + \left( {\frac{{\partial \alpha_{AB} }}{{\partial Y_{B} }}} \right)^{2} m_{{Y_{B} }}^{2} .$$

It is possible to ascertain the vertical angle to the laser scanner from each point in the scanned object. To ascertain the vertical angle from point A, given that the coordinates of the occupied laser scanner station are (0, 0, 0), the following method, as illustrated in Fig. 6, can be employed:

8 $$\gamma_{A} = \tan^{ - 1} \left( {\frac{{Z_{A} }}{{\sqrt {X_{A}^{2} + Y_{A}^{2} } }}} \right)$$

By using the propagation law, the accuracy of vertical angle can be determined as following:

9 $$m_{\gamma }^{2} = \frac{{(X_{A}^{2} Z_{A}^{2} )m_{{X_{A} }}^{2} + (Y_{A}^{2} Z_{A}^{2} )m_{{Y_{A} }}^{2} + (X_{A}^{4} + 2X_{A}^{2} Y_{A}^{2} + Y_{A}^{4} )m_{{Z_{A} }}^{2} }}{{(X_{A}^{2} + Y_{A}^{2} ).(X_{A}^{2} + Y_{A}^{2} + Z_{A}^{2} )^{2} }}$$

The horizontal and vertical angles between observed points were calculated for all nine TLS scans and compared with the corresponding angles derived from total station observations. The comparison indicates that the accuracy of both horizontal and vertical angles is strongly influenced by scanning distance and angular resolution.

For horizontal angles, the standard deviations obtained from TLS observations ranged between approximately ± 0.001° and ± 0.007°, while the corresponding RMSE values varied between ± 0.002° and ± 0.011°. The largest angular discrepancies were observed at the longest scanning distance (26.491 m) combined with the lowest angular resolution (0.035°). In contrast, the smallest RMSE values were obtained at a scanning distance of 14.878 m with a higher angular resolution of 0.007°.

These results indicate that increasing the scanning distance generally leads to larger angular uncertainties, while higher angular resolution significantly improves the precision of angular measurements derived from TLS observations.

## Determination of geometric parameters (exterior orientation) from TLS observations

After evaluating the geometric accuracy of TLS observations through coordinate, distance and angular analyses, this test was performed also to determine the geometric characteristics of the imaging and surveying system, which included both inside and outside orientation components of TLS. The Interior orientation parameters define the camera’s fundamental features, including focal length, primary point coordinates, and lens distortion coefficients. In contrast, the Exterior Orientation parameters determine the sensor’s spatial position and orientation, which are represented by three-dimensional coordinates (X, Y, Z) and rotation angles. To guarantee appropriate integration between image-based data and the Total Station coordinate system, transformation characteristics such as scale and translation were also taken into consideration^32,33^. In this section, the exterior orientation between total station and TLS are determined. The geometric parameters (exterior orientation) derived from TLS observations were determined using the three-dimensional coordinates extracted from the processed point cloud data. These parameters include the relative spatial positions of selected targets, the distances between structural points, and the geometric relationships defining the structural configuration. The obtained TLS-based geometric parameters were compared with reference measurements obtained from total station observations in order to evaluate the capability of TLS for accurate geometric analysis in engineering surveying applications.

It is known that the coordinates obtained by a scanner include systematic measurement errors. For example, these errors include distance measurement errors by TLS rangefinder, angle measurement errors and measurement errors under disturbing conditions (temperature, wind, pressure …etc.). In this paper, we propose the following methodology for a general approach for calibrating a terrestrial laser scanner and determining the values of systematic errors in distances and angle measurements obtained by the scanner. We write the initial equation for the transition from the scanner coordinate system to an external system as follows.

10 $$\left[ \begin{gathered} X_{i} \hfill \\ Y_{i} \hfill \\ Z_{i} \hfill \\ \end{gathered} \right]_{Total\;Station} = \left[ \begin{gathered} T_{X} \hfill \\ T_{Y} \hfill \\ T_{Z} \hfill \\ \end{gathered} \right] + R(\omega_{X} ,\omega_{Y} ,\omega_{Z} ) \cdot \left[ \begin{gathered} X_{i} \hfill \\ Y_{i} \hfill \\ Z_{i} \hfill \\ \end{gathered} \right]_{TLS.} ,$$

where $$\left[ \begin{gathered} X_{i} \hfill \\ Y_{i} \hfill \\ Z_{i} \hfill \\ \end{gathered} \right]_{Total\;Station.}$$—Coordinates of observed points resulted from total station.

$$\left[ \begin{gathered} T_{X} \hfill \\ T_{Y} \hfill \\ T_{Z} \hfill \\ \end{gathered} \right]$$—Displacement Vector; $R(\omega_{X} ,\omega_{Y} ,\omega_{Z} )$—Rotation matrix; $$\left[ \begin{gathered} X_{i} \hfill \\ Y_{i} \hfill \\ Z_{i} \hfill \\ \end{gathered} \right]_{TLS}$$—Coordinates of the same observed points resulted from TLS.

The rotation matrix between the two systems can be calculated using the following formula:

11 $$R(\omega_{X} ,\omega_{Y} ,\omega_{Z} ) = \left[ {\begin{array}{*{20}c} {R_{11} } & {R_{12} } & {R_{13} } \\ {R_{21} } & {R_{22} } & {R_{23} } \\ {R_{31} } & {R_{32} } & {R_{33} } \\ \end{array} } \right],$$

where:

12 $$\left. {\begin{array}{*{20}l} {R_{11} = \cos \;\omega_{Y} \;\cos \;\omega_{Z} ;} \hfill \\ {R_{12} = \cos \;\omega_{Y} \;\sin \;\omega_{Z} ;} \hfill \\ {R_{13} = - \sin \omega_{Y} ;} \hfill \\ {R_{21} = \sin \;\omega_{X} \sin \;\omega_{Y} \cos \;\omega_{Z} - \cos \;\omega_{X} \sin \;\omega_{Z} ;} \hfill \\ {R_{22} = \sin \;\omega_{X} \sin \;\omega_{Y} \sin \;\omega_{Z} - \cos \;\omega_{X} \cos \;\omega_{Z} ;} \hfill \\ {R_{23} = \sin \;\omega_{X} \cos \;\omega_{Y} ;} \hfill \\ {R_{31} = \cos \;\omega_{X} \sin \;\omega_{Y} \cos \;\omega_{Z} - \sin \;\omega_{X} \sin \;\omega_{Z} ;} \hfill \\ {R_{32} = \cos \;\omega_{X} \sin \;\omega_{Y} \sin \;\omega_{Z} - \sin \;\omega_{X} \cos \;\omega_{Z} ;} \hfill \\ {R_{33} = \cos \;\omega_{X} \cos \;\omega_{Y} ,} \hfill \\ \end{array} } \right\}$$

where ω_X_, ω_Y_,ω_Z_ – Rotation angles in the X, Y and Z directions respectively.

Therefore, the vector of adjusted coordinates of any observed point obtained by TLS will have the form:

13 $$\left[ \begin{gathered} X_{i} \hfill \\ Y_{i} \hfill \\ Z_{i} \hfill \\ \end{gathered} \right]_{TLS.} = \left[ {\begin{array}{*{20}l} {\left( {s_{i} + \Delta s} \right).\cos (\alpha_{i} + \Delta \alpha ).\cos (\gamma_{i} + \Delta \gamma )} \hfill \\ {\left( {s_{i} + \Delta s} \right).\sin (\alpha_{i} + \Delta \alpha ).\cos (\gamma_{i} + \Delta \gamma )} \hfill \\ {\left( {s_{i} + \Delta s} \right).\sin (\gamma_{i} + \Delta \gamma )} \hfill \\ \end{array} } \right],$$

where: s_*i*_—the measured slope distance between the scanner and the observed point; α_*i*_ and γ_i_—the measured horizontal and vertical angles; ∆s, ∆α, ∆γ—collimation systematic errors for distance, horizontal and vertical angles.

The measured slope distances, horizontal and vertical angles from TLS to any observed point coordinates (X, Y and Z) can be determined using the formulas:

14 $$\left. {\begin{array}{*{20}l} {S_{i} = \sqrt {X_{i}^{2} + Y_{i}^{2} + Z_{i}^{2} ;} } \hfill \\ {tg\;\gamma = \frac{{Y_{i} }}{{X_{i} }};} \hfill \\ {tg\;\alpha = \frac{{Z_{i} }}{{\sqrt {X_{i}^{2} + Y_{i}^{2} } }}.} \hfill \\ \end{array} } \right\}$$

Therefore, when substituting the coordinates values obtained using the scanner into Eq. (13); expression (10) can be written as follows:

15 $$\left[ \begin{gathered} X_{i} \hfill \\ Y_{i} \hfill \\ Z_{i} \hfill \\ \end{gathered} \right]_{Tax.} = \left[ \begin{gathered} T_{X} \hfill \\ T_{Y} \hfill \\ T_{Z} \hfill \\ \end{gathered} \right] + R(\omega_{X} ,\omega_{Y} ,\omega_{Z} ) . \left[ {\begin{array}{*{20}l} {\left( {s_{i} + \Delta s} \right).\cos (\alpha_{i} + \Delta \alpha ).\cos (\gamma_{i} + \Delta \gamma )} \hfill \\ {\left( {s_{i} + \Delta s} \right).\sin (\alpha_{i} + \Delta \alpha ).\cos (\gamma_{i} + \Delta \gamma )} \hfill \\ {\left( {s_{i} + \Delta s} \right).\sin (\gamma_{i} + \Delta \gamma )} \hfill \\ \end{array} } \right].$$

Expression (15) contains nonlinear functional equations for the observed angles, distances, and nine unknown parameters. As a result, the coordinates of every observed point acquired during measurements process by the scanner and total station can be entered into the system of equations. It is possible to rebuild the nine unknown parameters using the least-squares method. For a particular scan, the nine unknown parameters and their corrected values were found. The least-squares approach and MATLAB software programming were used to solve this problem. Table 4 displays the final results for each scanning option **.**

**Table 4:** Transformation parameters for each TLS scan at different distances and angular resolutions.

Parameter	Distance 14.848 m			Distance 20.714 m			Distance 26.491 m		
	0.0350°	0.0150°	0.0070°	0.0350°	0.0150°	0.0070°	0.0350°	0.0150°	0.0070°
TX, mm	− 3128.2	− 3185.5	− 3173.9	2373.8	2158.2	1921.7	6241.6	6310.7	6195.8
TY, mm	3405.1	3321.8	3346.6	938.85	646.24	320.51	− 3604.2	− 3487.6	− 3620.3
TZ, mm	1541.6	1555.2	1549.7	1494.8	1529.3	1598.1	1529.4	1644.3	1525.4
ωX, °	0.0069	0,0352	0.0286	0.2304	0.3023	0.3707	0.4516	0.4904	0.4125
ωY, °	0.6169	0.6058	0.6169	0.1010	0.1494	0.1989	− 0.1252	− 0.0372	− 0.1696
ωZ, °	14.8605	15.0603	14.9859	38.5269	38.994	39.4358	30.9915	30.8191	30.8539
∆s, mm	− 42,25	− 12.11	− 16.380	− 20.46	− 23.45	− 34.47	− 40.47	− 49.01	− 18.634
∆α, °	− 0.0534	0.0901	0.048	− 1.9066	− 1.3924	− 0.8478	0.0814	− 0.0725	0.1146
∆γ, °	0.0313	− 0.0347	− 0.0084	0.0085	− 0.0146	− 0.096	− 0.0196	− 0.1696	− 0.0684

Based on the comparison presented in Table 4, the following conclusions can be drawn:

- The a priori accuracy estimation formulas are highly consistent with the obtained results, with a difference of less than 15%;
- An improvement in laser scanning resolution is associated with greater accuracy in obtaining point coordinates;
- The Riegl LMS-Z420i TLS reaches its highest accuracy in the obtained results at a test object distance of about 20 m.

## Study the accuracy of terrestrial laser scanner observations for deformation monitoring

**Fig. 7:**
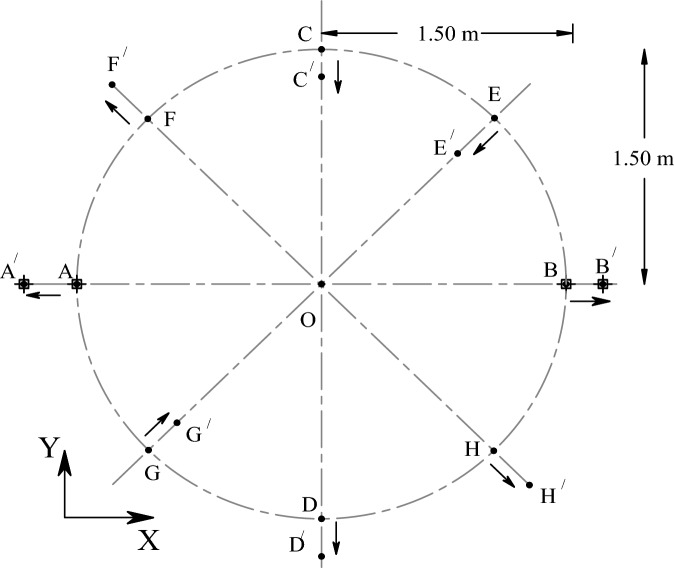
The distribution of points (reflective marks) for testing TLS observations in deformation monitoring.

Any special structure such as high rise building or circular oil tank may be anticipating small vertical or horizontal movements, frequently measured in millimeters or centimeters. To successfully and confidently detect exact and accurate point movement for these critical structures, it was necessary to assess the sensitivity and accuracy of the terrestrial laser scanner observations in detecting the deformation of deforming objects^34,35^. To achieve this purpose, the following laboratory test is performed. This experiment was conducted to evaluate the ability of using TLS to determine the values and directions of point’s displacements, given its capacity to perform a comprehensive three-dimensional scan of the structure. Nine reference points (reflective targets) were fixed on a vertical wall as sown in Fig. 7; eight of these points were distributed around the circumference of a circle with a radius of 1.5 m, while the ninth point was placed at the center of the circle.

**Fig. 8:**
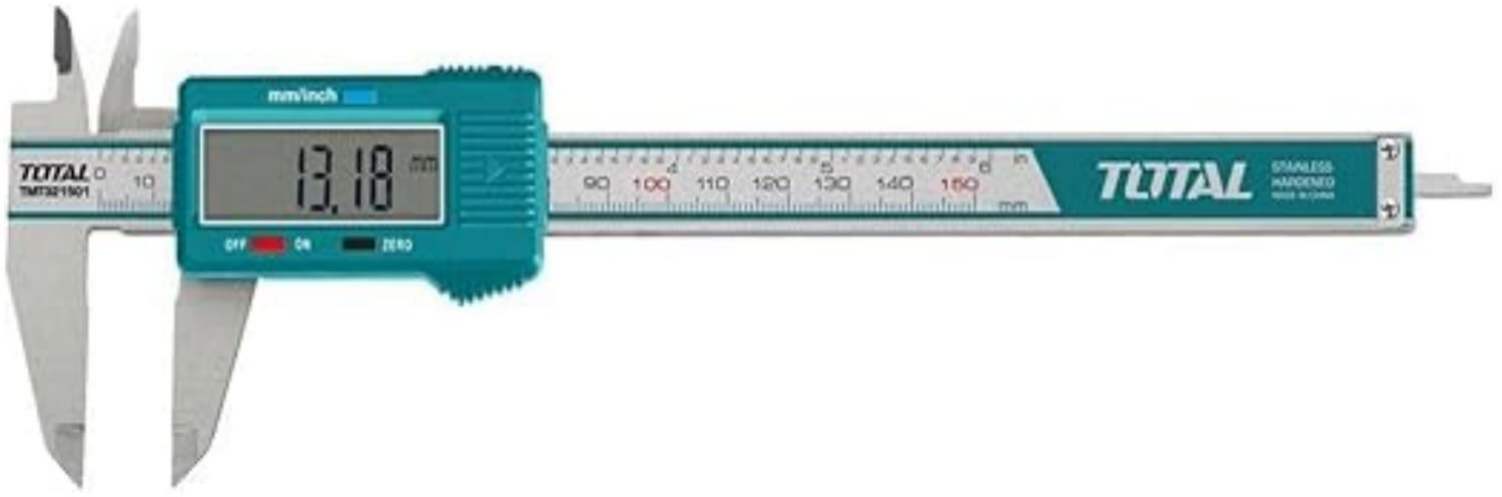
The used digital vernier in displacement measurements.

The main idea of the test is that the target at point O (circle center) will remain fixed (don’t move) while the other eight remarkable targets were moved such for points A and B be moved with proposed horizontal movements, but points C and D were moved in vertical movements but the other points (E, F, G and F) were moved in proposed inclined displacements as shown in Fig. 7 to simulate all the expected movements of any structure points and corners. A digital vernier (Fig. 8) with an accuracy of 0.02 mm was used to measure the displacements of all points in all directions accurately which will be considered as the reference measurement tool for calibrating the results of TLS observations when detecting the structure displacements. Marked target detection, registration, least-squares correction, and outlier filtering are critical processing processes completed prior to data analysis and processing, constituting a conventional and routine preprocessing workflow typically employed in terrestrial laser scanning applications.

The used TLS was positioned roughly 5.86 m away from the wall for all observations epochs (scans) and fixed with the same angular resolution for all scans and observations epochs to avoid any systematic errors. The TLS instrument performed two scans: the first scan was done to record the points’ initial positions of all remarkable marks and the second scan was done after the displacements. Minor and larger displacements values were applied to simulate the actual and real movements of structures. From the coordinates of all eight points in both scans, the displacements between each point and its new position after the movement and its accuracy were calculated. These values were then compared with the results of the digital vernier readings (with accuracy 0.02 mm). The results are presented in Table 5.

**Table 5:** Comparison of displacements values resulted from TLS observations and digital vernier.

Type of movements	Distance	Proposed movements, (mm)	Calculated distance from coordinates of TLS observations, mm			Measured distance by Vernier, mm	Discrepancies between distances, mm
			∆X	∆Y	D		
Horizontal	A–A^/^	110	− 111.2	–	111.2	110.42	0.78
	B–B^/^	45	+ 44.7	–	44.7	46.86	− 2.16
Vertical	C–C^/^	95	–	− 96.4	96.4	94.46	1.94
	D–D^/^	30	–	− 31.7	31.7	32.88	− 1.18
Slope	E–E^/^	85	− 61.4	− 61.8	87.1	85.22	1.88
	F–F^/^	70	− 50.1	+ 50.2	70.9	71.92	− 1.02
	G–G^/^	40	+ 29.7	+ 29.5	41.9	39.84	2.06
	H–H^/^	20	+ 14.0	− 14.1	19.9	21.34	− 1.44

Table 4 indicates that the discrepancies in measured displacements between the two points, simulated as deformation values, derived from the digital vernier and TLS are minimal. The maximum and minimum values for the difference are 2.16 mm and 0.78 mm, respectively. The results of this test clearly demonstrate that the terrestrial laser scanner, besides its primary benefit of enabling remote monitoring without physical contact with the target or structure, can accurately measure the displacements and movements in the monitoring of engineering structures such as circular steel oil tanks.

## Comprehensive investigation of vibration effects (dynamic conditions) on the accuracy of TLS observations

To achieve highly accurate results from terrestrial laser scanner observations during the process of structural deformation and inclination monitoring for engineering structures such as high-rise buildings, bridges, oil tanks, turbines … etc., the scanner must be stationary in relation to the scanned object. However, in many real field applications of structural deformation monitoring, the scanner is subjected to vibration from a variety of sources. Tripod vibrations, for example, can occur as a result of wind loads, vehicles, machinery, turbines or construction equipment movement.

As known, laser scanning technique is well recognized for finding the spatial coordinates of surface points by measuring distances between all visible points with a reflectorless laser rangefinder. The tripod-scanner system may experience minor translational and rotational perturbations as a result of these disruptions, which could affect angular observations and estimated distances. The findings show that angular measurements are more susceptible to these kinds of disturbances than range measurements.Errors induced by TLS vibration are often systematic in nature, affecting recorded angles and distances as well as the estimated coordinates of scanned points. Vibration has an impact on the geodetic instruments used to determine the displacements, settlement and structural deformation of engineering buildings. This paper investigates the effect of tripod vibration on the accuracy of TLS observations and scanned point coordinates.

**Fig. 9:**
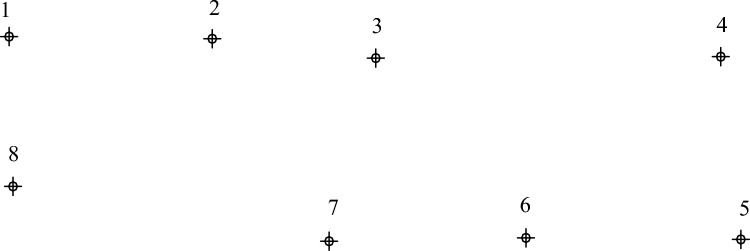
Distribution of observed points (marks) on wall to study the effect of tripod vibration on TLS observations.

In an elastic system, vibration is a mechanical oscillatory motion where at least one coordinates changes over time from increasing to decreasing. Unbalanced dynamic forces created by moving machinery, systems and transportation vehicles frequently cause vibrations. Therefore, studying the impact of tripod vibration on the accuracy and reliability of terrestrial laser scanning measurements is vital and necessary. To achieve this goal, an experimental study in this section was conducted. The experiment involved observing eight reflective markers and was conducted indoors under artificial lighting, at an ambient temperature of 20 °C and at normal pressure (Fig. 9).

**Fig. 10:**
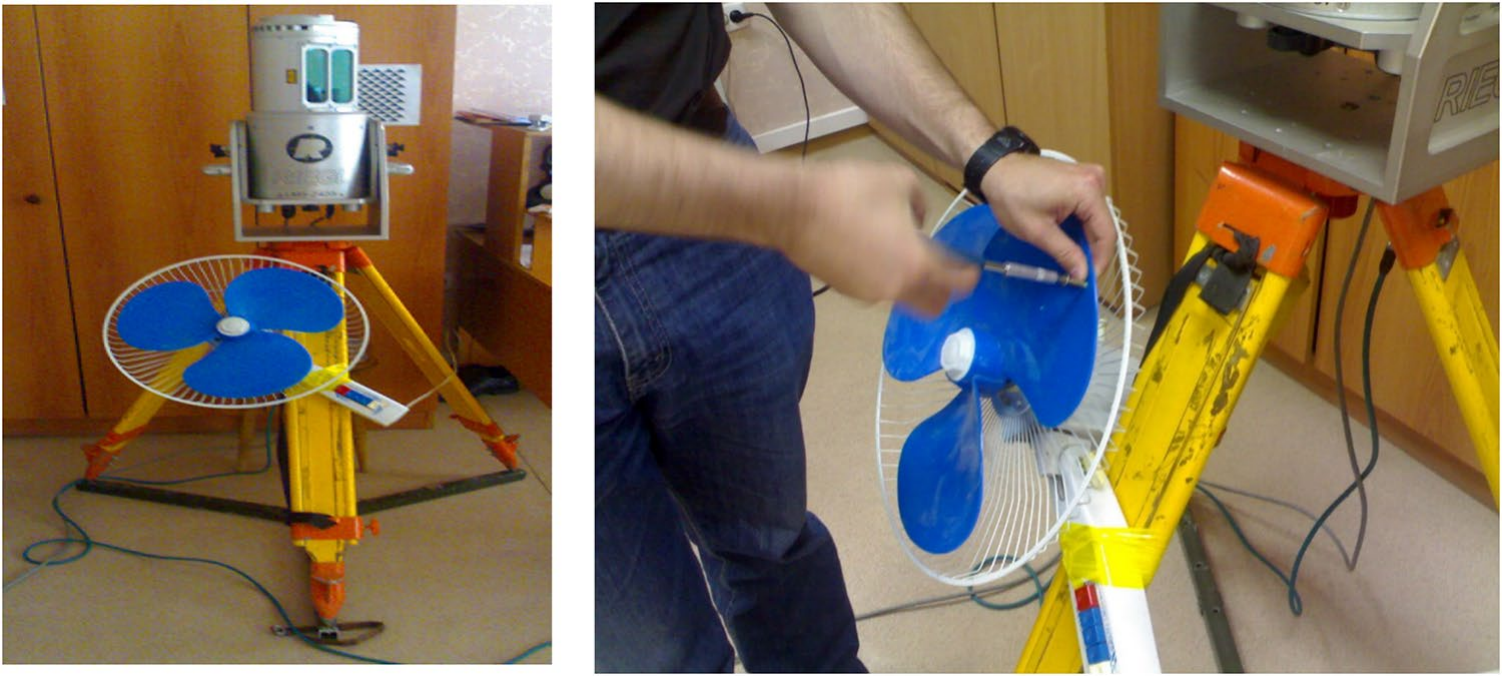
Simulation of tripod vibration and oscillation (dynamic conditions) to study their effect on TLS observations.

The coordinates of all points (marks) were determined at first with a Leica TCR 1205 PinPoint R100 total station, which has distance measurement precision 3 mm in reflectorless modewith a range of up to 170 m and has an angle measurement accuracy of 5.0". To simulate vibration (dynamic conditions), an electric fan was fixed to a tripod leg as shown in Fig. 10. To generate varied tripod oscillation and frequencies, an eccentric with extra mass was installed on the fan blade. Two tripod leg setups were completed. The fan was used as a controlled source to generate varying vibration levels for sensitivity assessment of TLS measurements, rather than for full spectral characterization of the tripod–scanner dynamic response.

The first setup used normal tripod legs with a 93 cm space between them (Fig. 10).The second arrangement lowered the space between the tripod legs to be 57 cm.

The experimental study was conducted using a Riegl LMS-Z420i terrestrial laser scanner. The test was done seven times (seven scans). The first scan was performed without the influence of tripod vibration. The remaining scans were obtained with different eccentric positions at three distances between the tripod legs. At each tripod leg position, scanning was performed with a different eccentric position. Initially, the fan was off when taking measurements (first scan). After that an eccentric mass was fixed to the fan blade at different three distances from the device’s axis of rotation and the measurements were repeated. Vibration data were captured concurrently with each scanning operation using a vibration measurement instrument that was firmly fixed to one of the tripod legs supporting TLS. Seven different scanning scenarios were carried out. When stimulation frequencies go close to the tripod-scanner system’s inherent frequencies, resonance can increase displacement and decrease measurement stability, which can have serious negative implications. A tri-axial accelerometer was used to record the vibration signals, and FFT-based vibration analysis was used to examine them in the frequency domain. The results are recorded in Table 5.

With dominating frequencies below 2 Hz and vibration amplitudes in the range of roughly 3 µm, the first scan was carried out under steady conditions without any induced vibration where the fan was off, showing minimal vibration levels. With the tripod leg distance kept at 93 cm, three further scans were performed with produced vibrations. Due to various stimulation configurations, increasing vibration levels were observed under these conditions, with dominating frequencies ranging from around 12 to 26 Hz and corresponding vibration amplitudes steadily increasing from roughly 8 µm to 22 µm. After lowering the tripod leg height to 57 cm, the remaining three scans were carried out. This resulted in a modified dynamic response of the support system, with dominant vibration frequencies varying between roughly 30 Hz and 48 Hz and vibration amplitudes ranging from 18 µm to 38 µm as shown in Table 6.

**Table 6:** Number, type and descriptions of all TLS scans for tripod vibration.

Distance between tripod legs	Scan number	Position description	Recorded vibration and oscillation values
93 cm	Position 0	Scan without vibration	2 Hertz–3 µm (can be neglected)
93 cm	Position 1	An eccentric mass was fixed to the fan blade at near axis of rotation	12 Hertz–8 µm
	Position 2	An eccentric mass was fixed to mid fan blade	20 Hertz–17 µm
	Position 3	An eccentric mass was fixed at fan blade end at the point furthest from axis of rotation	26 Hertz–22 µm
57 cm	Position 4	An eccentric mass was fixed to the fan blade at near axis of rotation	30 Hertz–18 µm
	Position 5	An eccentric mass was fixed to mid fan blade	40 Hertz–29 µm
	Position 6	An eccentric mass was fixed at fan blade end at the point furthest from axis of rotation	48 Hertz–38 µm

To study the effect of tripod vibration on the accuracy of TLS measurements, the following comparisons were made:

- Measured distances, horizontal and vertical angles and their accuracy from the scanner to the points (marks).
- The coordinates of observed points for all scans and its accuracy.
- The distances between the observed points, obtained by TLS for all tripod vibration values, and compare with total station observations.
- Calculated horizontal angles between the observed points (marks) and its accuracy for all scans.

Depending on the coordinates of the observed points for all TLS scans (seven scans), the distances, horizontal and vertical angles from the scanner to each point were determined for each scan. Based on the resulted distances and angles from the scanner to the observed points (marks), comparisons were made between the distances, horizontal and vertical angles obtained by the scanner with and without the influence of tripod vibration. Based on these comparisons, graphs were constructed showing the effect of tripod vibration on the measured distances and angles from the scanner to the points.

Based on the analysis of the results of distance and angle measurements from the scanner to the observed marks, including those affected by vibration, it is found that for all tripod vibration values, vibration causes changes in the measured distances from the scanner to the points, on average from 0.3 to 4.1 mm. The change in the measured horizontal angles obtained by the scanner, depending on the influence of tripod vibration, ranges from 4^//^ to 100^//^ with a relative error of (1:6000) to (1:8500). However, the change in the measured vertical angles can reach as much as 70^//^. Therefore, increased tripod vibration leads to increased TLS measurement errors and, consequently, a decrease in the accuracy of determining the coordinates of the points. It is noted also that errors in angle measurements from TLS observations due to the influence of tripod vibration are greater than errors in distance measurements. Tripod vibrations and oscillations, which simulates dynamic conditions, affects the spatial resolution of the laser scanner and, consequently, the obtained number of pixels covering the viewing area and leads to an error in the intensity of the images obtained by the scanner. Depending on the points coordinates for all scans, the discrepancies for all points coordinates are calculated where the first position without vibration is taken as a reference for all other scans. The results are tabulated in Tables 7 and 8.

**Table 7:** The differences of points coordinates during tripod vibration for tripod legs distance 93 cm.

Point	Discrepancies for position 1, mm			Discrepancies for position 2, mm			Discrepancies for position 3, mm		
	∆X = X_I_ – X_0_	∆Y = Y_I_ – Y_0_	∆Z = Z_I_ – Z_0_	∆X = X_2_ – X_0_	∆Y = Y_2_ – Y_0_	∆Z = Z_2_ – Z_0_	∆X = X_3_ – X_0_	∆Y = Y_3_ – Y_0_	∆Z = Z_3_ – Z_0_
1	0	− 1	− 1	1	− 3	− 2	1	− 5	− 2
2	0	− 2	− 1	0	− 4	− 1	1	− 4	− 2
3	− 1	− 3	0	− 1	− 5	− 1	− 2	− 9	− 1
4	− 1	1	0	− 1	− 2	− 1	− 2	− 3	− 1
5	− 1	0	− 1	− 1	− 3	0	0	0	− 2
6	− 1	− 2	− 1	− 1	− 3	− 1	− 1	0	0
7	− 1	− 2	0	0	− 2	− 2	− 1	− 4	− 1
8	0	− 2	− 1	1	− 4	− 2	1	− 6	− 2
Minimum	− 1	− 3	− 1	− 1	− 5	− 2	− 2	− 9	− 2
Maximum	0	1	0	1	− 2	0	1	0	*0*
Average	− 0.62	− 1.38	− 0.63	− 0.25	− 3.25	− 1.25	− 0.37	− 3.88	− 1.38
S. D	0.518	1.302	0.518	0.886	1.035	0.707	1.302	2.997	0.744

**Table 8:** The differences of points coordinates during tripod vibration for tripod legs distance 57 cm.

Point	Discrepancies for position 4, mm			Discrepancies for position 5, mm			Discrepancies for position 6, mm		
	∆X = X_4_ – X_0_	∆Y = Y_4_ – Y_0_	∆Z = Z_4_ – Z_0_	∆X = X_5_ – X_0_	∆Y = Y_5_ – Y_0_	∆Z = Z_5_ – Z_0_	∆X = X_6_ – X_0_	∆Y = Y_6_ – Y_0_	∆Z = Z_6_ – Z_0_
1	− 71	173	− 28	− 72	173	-28	− 71	172	− 27
2	− 39	176	− 24	− 39	174	− 25	− 38	174	− 25
3	− 14	176	− 24	− 13	173	− 22	− 13	174	− 22
4	45	176	− 15	46	177	− 16	46	175	− 18
5	39	164	− 20	40	165	− 23	41	168	− 20
6	5	169	− 25	4	167	− 24	5	165	− 24
7	− 29	170	− 22	− 28	172	− 23	− 28	168	− 22
8	− 80	169	− 30	− 78	167	− 28	− 79	166	− 31
Minimum	− 80	164	− 30	− 78	165	− 28	− 79	165	− 31
Maximum	45	176	− 15	46	177	− 16	46	175	− 18
Average	− 18.00	171.63	− 23.50	− 17.50	171.00	− 23.63	− 17.13	170.25	− 23.63
S. D	46.257	4.373	4.660	46.328	4.175	3.815	46.529	3.955	4.104

From Tables 6 and 7, the great effect of tripod vibration on the point coordinates and consequently the accuracy of TLS observations is clear. Based on the obtained results of the measured horizontal angles between the observed points, a comparison was made between the horizontal angles obtained by the scanner with and without vibration. The results of determining the deviations in the horizontal angles obtained between the marks using the Riegl LMS-Z420i scanner with and without vibration are presented in Table 9.

**Table 9:** Results of determining the deviations of horizontal angles by a scanner with and without the influence of vibration.

Horizontal angle between observed points	Discrepancies of horizontal angles by scanner with vibration, second α_vibration._ –α_without vibration_					
	Distance between tripod legs 93 cm			Distance between tripod legs 57 cm		
	Position 1	Position 2	Position 3	Position 4	Position 5	Position 6
α_1–2_	− 23	− 27	17	22	− 4	54
α_1–3_	− 51	− 53	− 107	− 17	− 74	12
α_1–4_	22	40	37	− 30	− 14	− 37
α_1–5_	− 7	15	121	18	− 96	− 121
α_1–6_	− 8	− 35	119	9	29	95
α_1–7_	24	− 28	18	1	76	33
α_1–8_	27	24	29	− 139	− 123	− 196
S. D., Second	28.8	35.1	76.8	56.0	71.3	103.4

From the analysis of the results presented in Table 8, it can be concluded that the tripod vibration has a great effect on the calculated horizontal angles between angles for all TLS scans.

Surveyors cannot control vibration parameters; they can only mitigate its impact on the measurement process. Several methods have been developed to mitigate the impact of vibration on the tripod-instrument system during measurements. The author of Blais^24^, proposes to reduce the impact of vibration on scanner measurements mathematically during data processing by using the Iterative Closest Point (ICP) algorithm. He suggests that the vibration of the scanned object can be considered instead of the vibration of the scanner, depending on the base; vibration is considered as the relative movement between the scanner and the object. This method proposes using an iterative scheme that begins with some initial estimate of the correct spatial arrangement of the source data and then refines this estimate using a procedure for minimizing the average distance between the two sets of ranging data.

A disadvantage of the proposed method is that measurement errors due to the large amplitudes of vibrations introduced during scanning remain. Another disadvantage is the need to perform this data processing method at each scanner setup, because the vibration frequency and amplitude vary with each setup, especially when scanning a wide area. In practice, the most effective method for mitigating the impact of tripod-TLS system vibration on measurements is to isolate the tripod-TLS system from the vibration source using external stabilization devices. This is accomplished by installing an additional device between the vibration source and the vibration protection object; these are typically vibration isolators, such as rubber shock absorbers. We developed a similar method for mitigating the impact of vibration on measurement errors of digital levels observations when determining deformations at nuclear power plants, state district power plants and thermal power plants^15,36^. The results showed that the use of vibration-isolating pads reduces the accuracy of elevation measurements of a high-precision level by only 5–15%^15^.

## Investigating the inclination of steel vertical oil tank using TLS

**Fig. 11:**
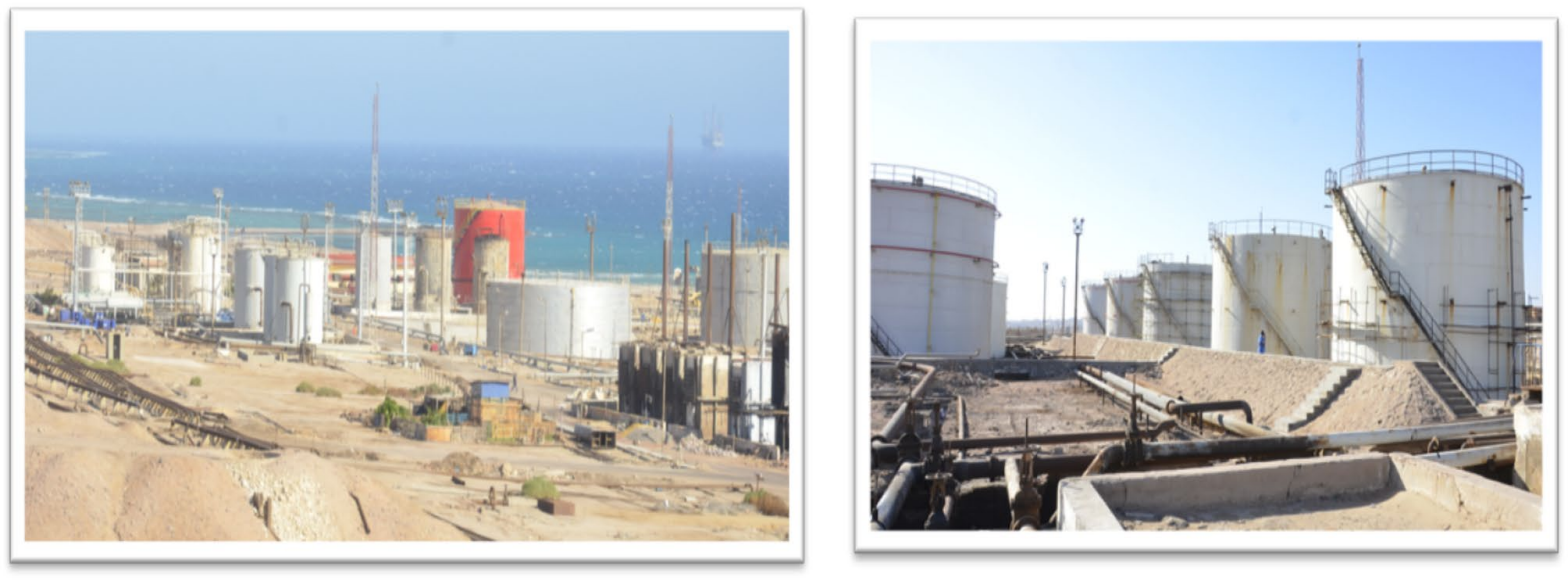
Oil tanks of Ras Gharib Plant, Egypt.

This section demonstrates a practical application of Terrestrial Laser Scanning in assessing the geometric properties and inclinations of a cylindrical vertical oil tank at the Ras Gharib Plant in Egypt. The Ras Gharib Plant is one of the 17 facilities operated by the General Petroleum Corporation (GPC), the premier national oil corporation in Egypt, the Middle East, and Africa, engaged in hydrocarbon exploration, production, and development. The site was assessed to identify impediments and hurdles that impede instrument operation, as well as to ascertain the scanning location (Fig. 11).

**Fig. 12:**
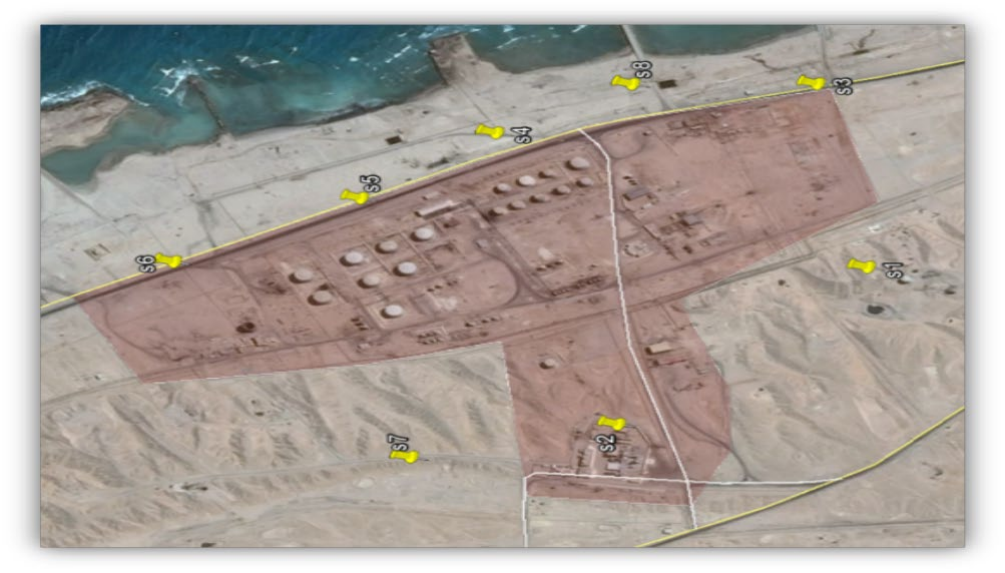
The occupied stations for plant scan using two different laser scanners.

The site was evaluated to detect obstructions and barriers that limit the operation of the instruments, as well as to determine the location for scanning. Two types of 3D laser scanners; the Optech Long Range and Faro Short Range laser scanners were utilized for data collection. Each scan took an average of six to seven minutes, and the total number of scans per day for the entire plant was twenty, which were completed in three and a half hours without accounting for changes in battery life, instrument transportation, or time after sunset. Eight positions around the pant are occupied to make a full scan for all parts of oil plant especially tanks as shown in Fig. 12.The processing of two tanks with various diameters was the main focus of this study.

The studied oil tank has approximately 23 m diameter and 12 m height and made of steel. The age of the tank, the geological formation of the soil around the tanks in this area, the uneven settlement of the tank foundations, the loading and unloading of oil and the temperature of the crude will all contribute to stress and strain on the tank’s membrane and sediment settlement. Radial distortion may be a common occurrence in the tanks. It is necessary to use an accurate geodetic methods and tools to monitor the inclination of this circular oil storage tank. Some operational acquisition details were unavailable due to confidentiality constraints connected with the externally provided industrial dataset. However, Table 10 now includes an overview of the possible field data acquisition parameters.

**Table 10:** Summary of field data acquisition parameters.

Parameter	Description
Study site	Ras Gharib Plant, Egypt
Scanner types	Optech Long Range and Faro Short Range TLS
Average scan duration	6–7 min per scan
Number of scans per day	20 scans
Total scanning time	Approximately 3.5 h
Number of scan positions	8 occupied stations
Survey coverage	Full coverage of the oil plant, especially storage tanks
Main analysis focus	Geometric assessment and inclination monitoring of two tanks
Studied tank dimensions	Approximately 23 m diameter and 12 m height
Potential deformation factors	Settlement, loading effects, soil conditions, and temperature variations

### Determination of the center coordinates and radius of a vertical cylindrical steel oil tank

There are several methods for determining the radius of the circular section of a vertical cylindrical tank. Some of these techniques rely on geodetic measurements and the simplest geodetic technique uses steel tapes. This is done based on measuring the perimeter of the circular section of the tank. The measured perimeter length is equal to 2πr, from which the radius r can be calculated. The accuracy of radius determination using precise taping typically ranges approximately from 1.5 to 5 mm^7^. The radius r of a vertical cylindrical tank can be calculated from TLS-derived coordinates using n surface points (where n > 3) located on the perimeter of the circular cross-section by applying the combined least squares method as follows:

Any observation point (*x*_*i*_, *y*_*i*_) located on the perimeter of the circular cross-section of the tank outer surface must satisfy the following circle equation:

16 $$\left( {x_{i} - x_{C} } \right)^{2} + \left( {y_{i} - y_{C} } \right)^{2} - r^{2} = 0$$

where: *x*_*i*_, *y*_*i*_ are the coordinates of any point i located on the perimeter of the circular section; *x*_*C*_, *y*_*C*_ are the circular cross section center coordinates; r is the radius of circular cross section.

The general combined method of least squares adjustment is applied for Eq. (16), where the parameters and observables cannot be separated. The following equation is the general linear form of Eq. (16) for n points derived from TLS observations^1^:

17 $$\mathop A\limits_{(n,u)} .\mathop X\limits_{(u,1)} + \mathop B\limits_{(n,m)} .\mathop V\limits_{(m,1)} + \mathop L\limits_{(n,1)} = \mathop 0\limits_{(n,1)}$$

where:

n—The number of equations, where n is the number of monitoring points distributed on the same cross section; u, the number of unknowns (parameters); in this study u equals 3; which include radius r and center coordinates X_C_, Y_C_; m, the number of observations; in this study we find that m = 2 n; A, the matrix of partial differentials of Eq. (17) with respect to unknowns (X_C_, Y_C_, r) respectively. B, The matrix of partial differentials of Eqs. (16) with respect to (X_1_, Y_1_, X_2_, Y_2_, …, X_n_, Y_n_) respectively; L, the vector of constant terms associated with each of Eq. (17); V, the observed coordinates residuals vector.

Then, the steps of the combined general least squares model are applied to determine the corrected values of the cross section radius and its center coordinates, as well as its associated accuracy. A MATLAB program was developed to estimate the radius and center coordinates for any circular section along the height of the vertical oil tank.

### Determination of the inclination of a circular oil tank from radius and center coordinates

The following steps present the suggested procedure for determining the inclination of the axis and walls of a vertical circular oil tank based on the radius and center coordinates of the circular cross-sections derived from TLS observations analysis:

1. To determine the true deformed shape of the tank axis and the inclination of the tank walls, the height of the oil tank is divided into multiple horizontal sections at intervals of one meter, for example. Consequently, thousands of point coordinates derived from TLS observations and covering the entire outer surface of the tank are included within each horizontal section.
2. The radius values r_j_ and the center coordinates (X_c_, Y_c_) for each section at 1 m intervals (for example) along the tank height are determined using the least squares method through specially developed MATLAB software programs based on the coordinates of the scanned points obtained from TLS observations.
3. The magnitude and direction of the oil tank axis inclination are determined by comparing the center coordinates of the circular cross-sections at successive heights with the center coordinates of the first circular cross-section of the vertical tank as follows:
  18 $$\left. {\begin{array}{*{20}l} {Q_{{X_{J} }} = {\mathrm{X}}_{{{\mathrm{C}}_{{\mathrm{J}}} }} - {\mathrm{X}}_{{{\mathrm{C}}_{1} }} ;} \hfill \\ {Q_{{Y_{J} }} = {\mathrm{Y}}_{{{\mathrm{C}}_{{\mathrm{J}}} }} - {\mathrm{Y}}_{{{\mathrm{C}}_{{1}} }} ,} \hfill \\ \end{array} } \right\}$$
  where:
  ${\mathrm{X}}_{{{\mathrm{C}}_{{\mathrm{J}}} }} ,{\mathrm{Y}}_{{{\mathrm{C}}_{{\mathrm{J}}} }}$, center coordinates of any circular cross section j along the tank height; ${\mathrm{X}}_{{{\mathrm{C}}_{1} }} ,{\mathrm{Y}}_{{{\mathrm{C}}_{1} }}$, center coordinates of the first section of the cooling tower.
4. The inclination of the oil tank wall can then be determined based on the inclination of the tank axis and the radius of the circular cross-section for each section along the tank height as follows:
  19 $$\left. {\begin{array}{*{20}l} {q_{{X_{J} }} = Q_{{X_{J} }} \pm ({\mathrm{r}}_{{\mathrm{j}}} - {\mathrm{r}}_{1} );} \hfill \\ {q_{{Y_{J} }} = Q_{{Y_{J} }} \pm ({\mathrm{r}}_{{\mathrm{j}}} - {\mathrm{r}}_{1} )} \hfill \\ \end{array} } \right\}$$
5. The actual deformed shape of the vertical oil tank axis and the inclination of the tank walls can then be derived from the results obtained in steps 3 and 4.

### Results and discussion of the oil storage tank deformation analysis

For the studied tank, the radius and center coordinates of all circular sections along the tank height, together with their corresponding accuracies, were determined as presented in Table 11.

**Table 11:** Geometric parameters of the studied vertical steel oil tank derived from TLS observations.

Height of oil tank section, m	Radius of cross section, m	Standard deviation of tank section radius, mm	Coordinates of the center of the tank cross-section, m		Standard deviation of center coordinates, mm	
	*r*	*m*_*r*_	*X*_*0*_	*Y*_*0*_	*m*_*x*_	*m*_*y*_
1	11.4551	2.3	119.9534	340.1976	2.5	2.4
2	11.4531	2.2	119.9499	340.1909	2.6	2.8
3	11.4495	1.7	119.9519	340.1839	2.2	2.4
4	11.4461	1.6	119.9567	340.1732	2.0	2.2
5	11.4400	1.4	119.9635	340.1641	1.9	2.0
6	11.4334	1.3	119.9718	340.1573	1.7	1.8
7	11.4327	1.2	119.9726	340.1541	1.6	1.6
8	11.4316	1.2	119.9757	340.1544	1.6	1.6
9	11.4292	1.1	119.9766	340.1462	1.4	1.5
10	11.4311	1.0	119.9749	340.1442	1.4	1.5
11	11.4260	1.3	119.9788	340.1380	1.3	1.4
12	11.4227	1.1	119.9858	340.1360	1.2	1.3

The data presented in Table 9 indicates that the geometric parameters of the tank derived from TLS observations meet the required accuracy. Based on these results, the inclination values and their directions along the X and Y axes can be determined. These values represent the horizontal displacement of the center coordinates of the upper j-th section relative to those of the lower section. In addition, the magnitudes and directions of the inclinations along the walls of the vertical oil tank were also computed.

**Fig. 13:**
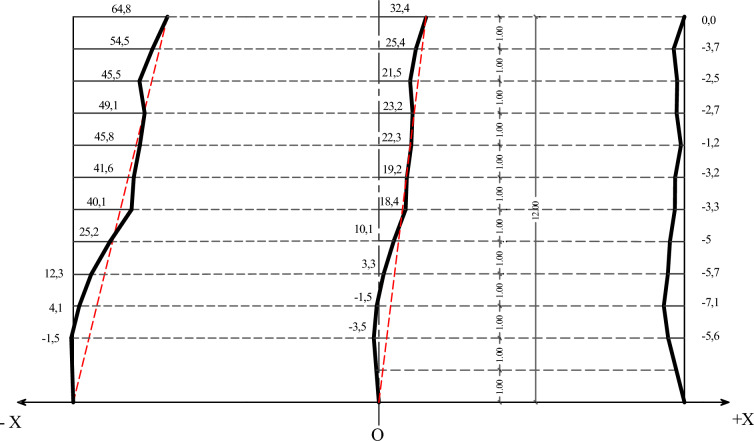
Inclination along the X-axis and walls of the studied circular oil tank from TLS observations analysis.

**Fig. 14:**
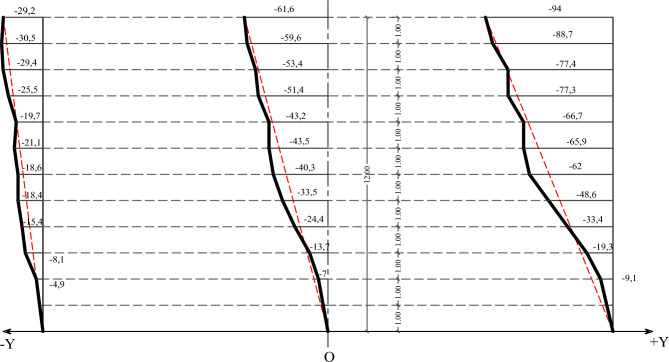
Inclination along the Y-axis and walls of the studied circular oil tank from TLS observations analysis.

Based on the obtained inclination magnitudes and directions for all tank sections, the inclination variations along the X and Y axes were plotted, together with the wall inclinations of the vertical steel oil tank (Table 12, Figs. 13, 14). These figures also illustrate the absolute deflections of the tank walls. The results confirm the high capability of using TLS for monitoring the geometric parameters of vertical circular tanks and determining the inclination of their axes and walls. The availability of spatial coordinates for a large number of measured points allows a highly detailed and accurate determination of these geometric parameters.

**Table 12:** Inclination values of the axis and walls each one meter along the height of the studied vertical cylindrical oil tank using TLS observations.

Height of oil tank section, m	Inclination values for tank axis in (X, Y) directions, mm			Inclination values for oil tank wall, mm			
	Q_X_	Q_Y_	Resultant (Q) $\sqrt {Q_{X}^{2} + Q_{Y}^{2} }$	q_x_		q_y_	
				+ X	–X	+ Y	– Y
1	0.0	0.0	0.0	0.0	0.0	0.0	0.0
2	− 3.5	− 6.7	7.6	− 1.5	− 5.5	− 4.7	− 8.7
3	− 1.5	− 13.7	13.8	4.1	− 7.1	− 8.1	− 19.3
4	3.3	− 24.4	24.6	12.3	− 5.7	− 15.4	− 33.4
5	10.1	− 33.5	35.0	25.2	− 5.0	− 18.4	− 48.6
6	18.4	− 40.3	44.3	40.1	− 3.3	− 18.6	− 62.0
7	19.2	− 43.5	47.5	41.6	− 3.2	− 21.1	− 65.9
8	22.3	− 43.2	48.6	45.8	− 1.2	− 19.7	− 66.7
9	23.2	− 51.4	56.4	49.1	− 2.7	− 25.5	− 77.3
10	21.5	− 53.4	57.6	45.5	− 2.5	− 29.4	− 77.4
11	25.4	− 59.6	64.8	54.5	− 3.7	− 30.5	− 88.7
12	32.4	− 61.6	69.6	64.8	0.0	− 29.2	− 94.0

## Conclusions

This research presents a comprehensive experimental, analytical and practical investigation of the geodetic precision of the Riegl LMS-Z420i terrestrial laser scanner for structural deformation monitoring under both static and dynamic conditions. The study includes theoretical accuracy propagation, controlled laboratory experiments, vibration analysis and a real-world industrial case study to assess the reliability and limitations of terrestrial laser scanning (TLS) in high-precision engineering applications. From the results, the following conclusions should be drawn:

1. The experimental comparison of TLS observations with total station observations indicates that TLS can achieve millimeter-level accuracy in coordinate determination, distance measurement, and angle estimation. Although total station observations provide superior horizontal coordinate precision, TLS demonstrated consistent and stable performance with coordinate accuracy often within approximately 40% of that achieved by total station measurements.
2. The investigation confirmed that both scanning distance and angular resolution significantly affect the TLS observations accuracy. Higher angular resolution improves coordinate and angular accuracy; however, increasing the scanning distance leads to a systematic degradation of the results. The optimal performance of the scanner was achieved at an object distance of approximately 20 m.
3. The deformation monitoring experiment confirmed the capability of using TLS to detect horizontal, vertical and inclined displacements and movements with discrepancies ranging between 0.78 and 2.16 mm when compared with high-precision digital vernier measurements. These findings demonstrate that TLS provides sufficient sensitivity for monitoring the inclination and displacements in engineering structures such as circular buildings, oil and water vertica; tanks, chimneys, cooling towers and high-rise buildings, while maintaining the advantage of remote and non-contact measurements.
4. The comprehensive vibration analysis revealed that tripod-induced oscillations significantly affect TLS measurements. Distance errors increased up to 4.1 mm, while angular deviations reached approximately 100 arc-seconds under several vibration conditions. The results indicate that angle measurements are more sensitive to vibration than distance measurements and that the stability of the support system is essential for maintaining measurement accuracy. Therefore, effective vibration isolation and stabilization techniques are crucial and necessary when using TLS in dynamic or industrial environments.
5. The practical application conducted at the oil storage facility of the General Petroleum Corporation in Ras Gharib city, Egypt, further demonstrated the applicability of TLS for real-world structural health monitoring. The inclination and geometric parameters of vertical cylindrical steel oil tanks were accurately determined using Optech Long Range and FARO Focus laser scanning systems through least-squares circle fitting and spatial mathematical modeling. The real case study confirmed that TLS provides an efficient, rapid and reliable approach for detecting geometric deviations and inclinations in critical infrastructure.

Finally, the results of this research demonstrate that terrestrial laser scanning is a powerful and reliable geodetic tool for monitoring the structural deformation and inclination, provided that appropriate scanning parameters are selected and environmental conditions (static and dynamic) are properly managed. Although TLS does not completely replace traditional total station measurements in high-precision applications, it offers significant advantages in terms of measurement speed, data density, three-dimensional completeness, and remote operation. The findings of this study provide practical guidance for selecting scanning configurations, understanding accuracy limitations, and mitigating vibration effects, thereby supporting informed decision-making in structural health monitoring and accurate engineering surveying applications.

## Data Availability

All data, models, and code generated or used during the study appear in the submitted article.
